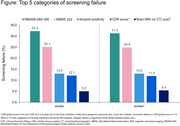# Reasons for screening failure in the evoke and evoke+ trials of semaglutide for early AD

**DOI:** 10.1002/alz.088735

**Published:** 2025-01-09

**Authors:** Mary Sano, Jeffrey L. Cummings, Howard H. Feldman, Oskar Hansson, Wiesje M. van der Flier, Lars Bardtrum, Rose Jeppesen, Peter Johannsen, Teresa Léon, Charlotte T. Hansen, Philip Scheltens

**Affiliations:** ^1^ Icahn School of Medicine at Mount Sinai, New York, NY USA; ^2^ James J Peters VAMC, Bronx, NY USA; ^3^ University of Nevada, Las Vegas, NV USA; ^4^ University of California San Diego, La Jolla, CA USA; ^5^ Lund University, Lund Sweden; ^6^ Skåne University Hospital, Malmö, 21428 Skåne Sweden; ^7^ Vrije Universiteit Amsterdam, Amsterdam, 1081 HV Netherlands; ^8^ Novo Nordisk A/S, Søborg, 2860 Søborg Denmark; ^9^ EQT Life Sciences Partners, Amsterdam, 1071 DV Amsterdam Netherlands

## Abstract

**Background:**

Alzheimer’s disease (AD) trials report a high screening failure rate (potentially eligible trial candidates who do not meet inclusion/exclusion criteria during screening) due to multiple factors including stringent eligibility criteria. Here, we report the main reasons for screening failure in the 12‐week screening phase of the ongoing evoke (NCT04777396) and evoke+ (NCT04777409) trials of semaglutide in early AD.

**Method:**

Key inclusion criteria were age 55‐85 years; mild cognitive impairment due to AD (Clinical Dementia Rating [CDR] global score of 0.5 plus CDR of ≥0.5 in ≥1 of three activities of daily living categories) or mild AD dementia (CDR global score of 1.0); Repeatable Battery for the Assessment of Neuropsychological Status delayed memory index (RBANS‐DMI) score ≤85; Mini‐Mental State Examination (MMSE) score ≥22; amyloid positivity (confirmed by positron emission tomography or cerebrospinal fluid). Additionally, evoke+ allowed the inclusion of participants with significant small vessel pathology (>1 lacunar infarct and/or age‐related white matter changes >2 [white matter >20 mm]). Among the exclusion criteria were evidence of neurological disorders other than AD; evidence of a clinically relevant or unstable psychiatric disorder; magnetic resonance imaging (MRI)/computerized tomography (CT) scan suggestive of clinically significant structural central nervous system (CNS) disease (other than the changes allowed for evoke+) nor suggestive of strategic infarcts. Screening failure rates were summarized descriptively. Data cleaning of the 3806 randomized individuals is ongoing in evoke/evoke+.

**Result:**

The total number of screening failures was 2,836 (58.6%) in evoke and 3,087 (59.8%) in evoke+. The top five categories of screening failure in evoke and evoke+ were RBANS‐DMI score ≤85, MMSE score ≥22, amyloid positivity, CDR score, and MRI/CT scan suggestive of CNS disease (other than the changes allowed for evoke+) or strategic infarcts (Figure). This reflected the hierarchical procedure applied during screening, whereby individuals were firstly assessed on cognitive scores, followed by exclusion of other neurological disorders and, lastly, confirmation of amyloid positivity.

**Conclusion:**

The hierarchical screening procedure in evoke and evoke+ resulted in most ineligible patients being excluded early in the screening process. The main reasons for screen failure were cognitive assessment scores.